# Effect of Health Model‐Based Menopausal Self‐Care Training on Quality of Life and Health: A Randomised Controlled Trial

**DOI:** 10.1111/hex.70511

**Published:** 2025-11-28

**Authors:** Khadijeh Khademi, Mohammad Hossein Kaveh, Mahin Nazari, Abdolrahim Asadollahi

**Affiliations:** ^1^ Student Research Committee, School of Health, Shiraz University of Medical Sciences Shiraz Iran; ^2^ Research Center for Health Sciences, Institute of Health, Department of Health Promotion, School of Health Shiraz University of Medical Sciences Shiraz Iran; ^3^ Department of Health Promotion, School of Health Shiraz University of Medical Sciences Shiraz Iran; ^4^ Department of Gerontology, School of Health Shiraz University of Medical Sciences Shiraz Iran

**Keywords:** community‐based participatory research, empowerment, menopause, quality of life, self‐care, women's health

## Abstract

**Background:**

Menopause is a natural biological transition in a woman's life, often accompanied by various physical and emotional challenges. Enhancing postmenopausal women's health and quality of life requires empowerment, particularly through self‐care strategies. The present study aimed to evaluate the effectiveness of a training programme based on Longwe's Empowerment Framework and a Community‐Based Participatory Research approach.

**Methods:**

A randomised controlled trial was conducted involving 140 postmenopausal Iranian women, with 70 women in each control and intervention group. The intervention group received a 6‐week training programme, with weekly 120‐min sessions, designed using Longwe's framework in combination with the PRECEDE‐PROCEED model. Data were collected at three time points: baseline, 2 weeks post‐intervention and 4 months post‐intervention, using validated questionnaires. Statistical analyses were performed using SPSS version 27, with significance set at *p* < 0.05.

**Results:**

The training intervention significantly improved participants' quality of life (η² = 0.23, *p* < 0.001) and overall health (η² = 0.12, *p* < 0.001), with large and moderate effect sizes, respectively. Notably, the programme had a large effect on menopausal self‐care behaviours (η² = 0.42, *p* < 0.001) and a small but significant effect on internal health locus of control (η² = 0.05, *p* < 0.001).

**Conclusion:**

This study demonstrates that a structured self‐care training programme, based on empowerment theory and community engagement, can effectively enhance the health outcomes and quality of life of postmenopausal women. The findings indicate that integrating participatory and empowerment‐based educational interventions into public health initiatives for midlife women is advantageous.

**Patient or Public Contribution:**

This study employed a community‐based participatory research approach, actively engaging postmenopausal women as collaborators throughout the intervention process. Participants were involved in the initial needs assessment, where they shared their self‐care priorities and preferences. They also provided input on the structure, scheduling, and delivery format of the educational sessions. Furthermore, they collaborated to develop and participate in weekly group activities that promoted self‐care behaviours. Ongoing feedback from participants was incorporated into refining of the intervention, improving its cultural relevance, feasibility and alignment with the lived experiences of the target population.

**Abbreviations**
13‐item MHLC‐C: Multidimensional Health Locus of Control Scale form C for menopausal women,
MSCQ: Menopause Self Care Questionnaire,
WHOQOL‐ BREF: World Health Organization Quality of Life,
WHQ: Women Health Questionnaire.

## 1 Introduction

Menopause is a natural biological process in women's lives. The average age of menopause for women worldwide is 52 years, with a range of 40–58 years [35, 41]. This means that one‐third of women's lives are spent in the postmenopausal period [15]. Due to various health concerns, this population group has a priority in maintaining and improving their health [29].

Vasomotor issues affect 85% of postmenopausal women, while sexual function disorders affect 87% of them. Osteoporosis impacts 55% of women in this group, and depression affects 20%–30% of them. Cardiovascular diseases are responsible for 45% of deaths in postmenopausal women [5, 7, 30]. Menopausal symptoms and health problems contribute to an increase in disease burden and treatment costs [34]. In many societies, fertility is seen as a symbol of youth and attractiveness. Consequently, menopause can lead to negative attitudes towards women going through this stage of life [3, 27, 40].

Research on menopause has shown that the quality of life for women tends to decrease, making it a significant issue in global health [21, 38]. Behavioural factors have been identified as determinants of postmenopausal health and quality of life. These include practicing self‐care behaviours, seeking adequate health care, and adopting a healthy lifestyle [10, 12].

Self‐care involves making lifestyle changes and prioritising one's health. Taking small daily actions in this area can have numerous positive effects on health [8, 28]. This highlights the importance of emphasising the ability to change and modify these behaviours through practical training, with empowerment being the most crucial aspect [19, 36].

Empowerment is defined as a process through which individuals gain control over decisions and actions affecting their health and determinants. In this way, empowerment can enhance an individual's sense of control by encouraging them to take responsibility for modifying their behaviour and overcoming any environmental obstacles [13, 19, 36]. Longwe's empowerment framework specifically examines, analyses, and promotes three levels of importance for women in a hierarchical manner: individual, interpersonal, and societal [13]. This framework consists of five levels: equity (access to social‐environmental facilities and services regardless of gender, ethnicity, etc.), access (possibility of using social‐environmental facilities and services), awareness raising (information related to health, facilities and social‐environmental services), participation (identifying health needs, taking action to solve them and using social‐environmental facilities and services) and control (decision‐making and actions to achieve maximum health, social‐environmental facilities and services) [20]. However, studies have primarily focused on modifying knowledge and attitudes alone, without addressing structural empowerment, which may be ineffective and has contributed to the inefficacy of current care‐service programmes aimed at improving the health and quality of life of postmenopausal women. Therefore, a population‐based approach can be utilised [27].

The community‐based participatory approach actively engages the community and has the potential to reduce health inequalities. This method is particularly effective when combined with the ecological model, which considers individual needs [12, 17]. The PRECEDE‐PROCEED model serves as an ecological and community‐based framework for health promotion, guiding needs assessment, development of tailored intervention programmes, and their implementation and evaluation. Thus, it acts as a roadmap for health initiatives [2, 37]. In contrast, research studies that focused solely on education or counselling has shown limited success in this area [26, 32].

This study aimed to enhance understanding of how to effectively promote the quality of life, health and self‐care of postmenopausal women. It sought to utilise the PRECEDE‐PROCEED model as a road map for a community‐based participatory approach and Longwe's empowerment framework as a guideline for women's empowerment in self‐care. Additionally, the study aimed to investigate the impact of empowerment training based on using both models simultaneously on the quality of life, health and self‐care of postmenopausal Iranian women.

## 2 Materials and Methods

### 2.1 Study Design and Population

This randomised controlled trial with a parallel design was conducted in Shiraz, Iran, from October 2023 to May 2024. The sample size of 134 participants was calculated based on the mean ± SD difference of the quality of life score in the empowerment intervention group from a similar study [17], using NCSS‐PASS version 15.0.13 with a type I error rate of 0.05%, a test power of 90%, and a 20% attrition rate. Ultimately, 150 women were included in the study to ensure an equal number of participants in each group and sub‐group (75 and 25 women, respectively).

**Figure 1:**
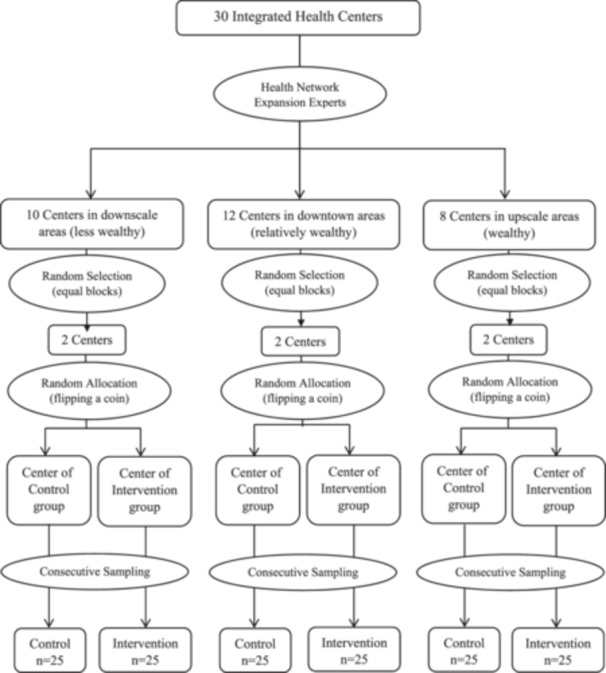
Sampling process of study.

Participants were recruited using multistage cluster sampling and randomisation based on Longwe's empowerment framework, specifically focusing on the construct of equity [20]. Two control and intervention groups were created to ensure demographic similarity. The integrated health centres of Shiraz City were selected as clusters in the present study using the NCSS‐PASS version 15.0.13 software (Figure 1).

**Figure 2:**
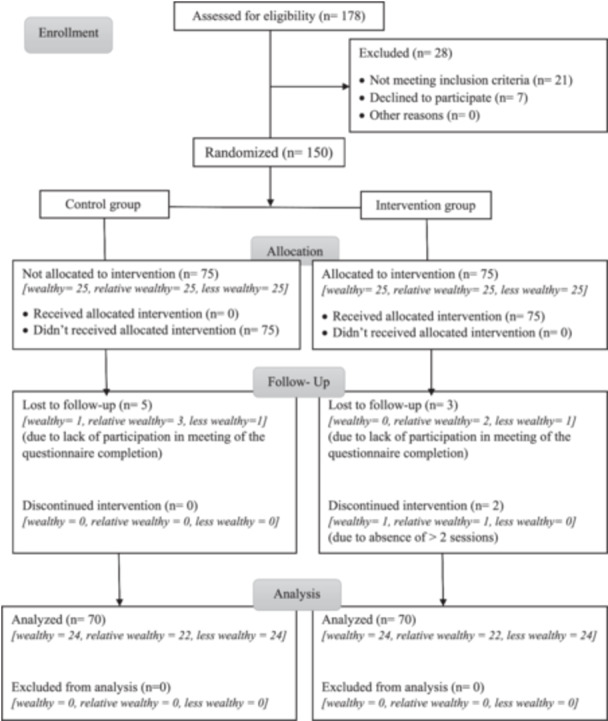
The CONSORT diagram of the study. CONSORT, Consolidated Standards of Reporting Trials.

Inclusion criteria included: women aged 45–65 years, who had experienced natural menopause at least 1 year prior, had a minimum literacy level equivalent to the third grade of elementary education, were not undergoing psychiatric treatment for mental illnesses, did not have chronic diseases such as diabetes, hypertension, etc., had not used hormone replacement therapy in the past 6 months, had no history of hysterectomy or oophorectomy surgery, and had not experienced abnormal vaginal bleeding. Exclusion criteria included: missing more than two sessions, failing to participate in the pre‐test, Posttest, or follow‐up test; taking hormone therapy, undergoing hysterectomy or oophorectomy surgery, experiencing abnormal vaginal bleeding, developing various types of cancer during the study, or being unwilling to continue participation. These criteria have a significant impact on the quality of life and health of women [5, 7, 30, 35]. Data were analysed using a per‐protocol approach, including only participants who did not meet the exclusion criteria to ensure a valid estimation of the intervention effect among adequately exposed participants [1]. Figure 2 displays the study's CONSORT (*Consolidated Standards of Reporting Trials*) diagram.

### 2.2 Data Collection

To implement a needs assessment approach using the PRECEDE‐PROCEED model, participants were required to fill out five questionnaires: the Persian version of the short form of the World Health Organization Quality of Life (WHOQOL‐BREF) [39], the Women Health Questionnaire (WHQ) [11], the Menopause Self‐Care Questionnaire (MSCQ) [16] the Multidimensional Health Locus of Control Scale form C for menopausal women (13‐item MHLC‐C) [18], and a questionnaire regarding demographic and reproductive characteristics.

The WHOQOL‐BREF consists of 26 questions rated on a 5‐point Likert scale. This questionnaire is categorised into physical health, mental health, social relations, environment, and overall quality of life. Each category can receive a score between 4 and 20, which is converted to a scale of 0–100 [23]. In Iran, the Intraclass Correlation Coefficient (ICC) was reported to be above 0.70 [39].

The WHQ consists of 36 questions using a 4‐point rating scale across nine dimensions: depressed mood, physical symptoms, anxiety/fear, vasomotor symptoms, sleep problems, sexual behaviours, menstrual symptoms, memory/concentration, and attractiveness. The total score is converted from 0 to 9. It should be noted that depending on the women's conditions, the dimensions of menstrual and sexual symptoms mentioned in the WHQ questionnaire cannot be used. All correlation coefficients of the validation process were greater than 0.75 [14]. The Cronbach's alpha and ICC coefficients of the Persian version of this questionnaire in Iran were 0.79 and 0.71, respectively [11]. In the present study, the dimension of menstrual symptoms was not included, so the range of women's health scores was reported on a scale of 0–8.

The MSCQ is an original Persian tool consisting of 33 questions using a 5‐point Likert scale in seven dimensions: general self‐care, screening, nutrition, memory enhancement, hot flashes and night sweats, sexual desire, and social communication. The total scores range from 33 to 165. Cronbach's alpha and ICC coefficients were 0.76 and 0.88, respectively [16].

For the first time, the validity and reliability of the Persian version of the 18‐item MHLC‐C scale were established among pregnant women. Cronbach's alpha coefficient ranged from 0.62 to 0.90 for these factors [25]. In a study involving menopausal women, Cronbach's alpha and McDonald's Omega coefficients for the 13‐item MHLC‐C scale were 0.81 and 0.72, respectively [18].

The demographic and reproductive characteristics were selected because evidence suggests they play a role in determining the health and quality of life of postmenopausal women [6, 9, 25, 31, 34, 38].

Pre‐test data, also referred to as pre‐intervention data and needs assessment, were collected from the study's instruments along with demographic information 1 week before the training sessions began on the introductory day. Posttest data, also known as post‐intervention data, were gathered 2 weeks after the intervention ended. Follow‐up test data were collected 4 months later. Both posttest data and follow‐up test data were considered for impact and outcome evaluation.

### 2.3 Procedure and Intervention

The first step in developing a community‐based programme involves conducting a needs assessment, for which the PRECEDE‐PROCEED model is considered the most effective framework. This model consists of three main phases: (1) assessing needs – covering social, epidemiological (including behavioural factors), educational‐ecological, and administrative‐policy dimensions; (2) designing an intervention based on these assessed needs; and (3) evaluating the implemented intervention [2, 37]. In this study, the needs assessment phase evaluated various components: quality of life (as part of the social assessment), health and self‐care (as part of the epidemiological assessment), and health locus of control (as part of the educational assessment). These variables were examined to guide both the needs assessment and the evaluation of the intervention's outcomes and impacts. Findings from the assessment indicated a clear need for intervention across all variables. Furthermore, an administrative and policy review was conducted through a literature survey 3 months before implementing the intervention. This review highlighted consistent recommendations across multiple studies emphasising the importance of empowering women [12, 19, 36]. Based on these insights, the intervention was designed using Longwe's empowerment framework [20], as described below:

#### 2.3.1 Equity, Access, and Awareness

To promote the programme titled ‘*Empowerment in Health Care for Postmenopausal Women’,*two posters were placed in each participating health centre 2 months before the training sessions. Additionally, bulk SMS messages outlining the training programme were sent to postmenopausal women registered with these health centres 1 month before the sessions. Two weeks before the training began, phone calls were also made to personally inform eligible women about the course and guide them through the registration process.

#### 2.3.2 Equity, Access, and Participation

In the first session, participants formed small groups and selected group leaders. Together, they identified self‐care priorities based on their own perspectives. The timing and structure of the training sessions were customised to suit the women's preferences. To facilitate continued interaction, virtual groups were created on WhatsApp for participants in specific health centres. Additional details can be found in Table 1.

**Table 1:** The topics as well as training methods and construction's focus of each training session in each selected health centre.

Session	Topic	Training methods	Intended constructs
1	❖ Reintroducing the educator, the subject of education, and its goals for women. ❖ Grouping women and selecting a group leader based on the women's choice (5 women in each group). ❖ Presenting the results of the pre‐test on quality of life, women's health, self‐care knowledge, and behaviours, highlighted the need for further efforts to improve these conditions. ❖ Identifying priorities for self‐care behaviours by comparing women's perspectives with those of the study team. ❖ Establishing the timing, day, frequency, and duration of training sessions based on women's preferences.	– Group discussion – Forming a virtual group on WhatsApp Messenger for sharing information, asking questions, and providing answers – ‘Menopausal Self‐care’ booklet	▪ Equity ▪ Accesses ▪ Participation
2	❖ Menopause: symptoms and management Definition, physiology, and aetiology of menopause Hot flashes and night sweats Fatigue and sleep problems Urinary, genital, and sexual problems Mood changes Weight gain Skin changes Common diseases (osteoporosis, heart diseases and cancers)	– Interactive lecturing Homework 1 – Implementing strategies throughout the week to alleviate menopause symptoms. – Filling out the weekly table of strategies for reducing menopause symptoms.	▪ Awareness ▪ Participation
3	❖ Physical activity Definition of physical activity Importance of physical activity Types of physical activity Duration of physical activity Preparation for physical activity	– Interactive lecturing – Group discussion About barriers to implementing strategies to reduce menopause symptomsHomework 2 – Complete Homework 1. – Engage in physical activity throughout the week. – Fill out the weekly table of physical activity.	▪ Awareness ▪ Participation ▪ Control
4	❖ Healthy nutrition Definition of healthy nutrition Importance of healthy nutrition Definition of Food pyramid and groups Definition of food unit for each food group	– Interactive lecturing – Group discussion Barriers to engage in physical activity and strategies for reducing menopause symptoms. Homework 3 – Complete Homework 1, 2. – Focus on healthy nutrition throughout the week. – Fill out the weekly table of healthy nutrition.	▪ Awareness ▪ Participation ▪ Control
5	❖ Stress Control (*focus on requesting social support)* Definition and physiology of stress Importance of social support in reducing stress Definition of social support Types of social support Strategies for requesting social support when needed	– Interactive lecturing – Group discussion Barriers to achieving healthy nutrition, engaging in physical activity, and managing menopause symptoms. Homework 4 – Complete Homework 1, 2, and 3. – Seeking social support throughout the week. – Fill out the weekly table of social support.	▪ Awareness ▪ Participation ▪ Control
6	❖ Post‐menopausal screening and examination Definition of screening Importance, time, and requirements of cervical screening (Pap smear and HPV test) Importance, requirements, time, and manner of breast screening (mammography, breast exam, and breast self‐exam) Importance, requirements, time of stool test, Bone Mineral Densitometry (BMD), checking blood pressure, calculating Body Mass Index (BMI), haematology test, urine test, oral examination, optic examination, and depressive screening.	– Interactive lecturing – Group discussion About barriers to receiving social support, maintaining a healthy diet, engaging in physical activity, and implementing strategies to reduce menopause symptoms.	▪ Awareness ▪ Participation ▪ Control

#### 2.3.3 Awareness, Participation, and Control

The intervention group participated in weekly, in‐person self‐care training sessions that were specifically designed for postmenopausal women. Each session lasted 120 min, with a 15‐min break included, and continued for six consecutive weeks. Starting from the third session, the first 45 min were dedicated to interactive education delivered by the primary researcher. Women were encouraged to choose their own methods of behaviour implementation based on scientifically valid sources. The instructional content was taken from a ‘Menopausal Self‐care’ booklet that followed the guidelines from Iran's Ministry of Health [24].

The following 60 min of each session were dedicated to group discussions that used a problem‐solving approach. During this time, participants shared feedback and reflected on self‐care behaviours they had practiced over the previous week. Table 1 provides a breakdown of each session's topics, teaching methods, and the behavioural constructs targeted.

Moreover, from session two through the follow‐up assessment (approximately 5 months later), each group leader, in collaboration with their subgroup, organised weekly outings to parks or green spaces. These gatherings included physical activity and the consumption of healthy foods. As an incentive, the five most active participants from each centre (15 women in total) received awards.

In the control group, no interventions were performed. However, participants did receive usual care and attended two educational sessions (totalling 2 h) on the topics of ‘correct use of medicine’ and ‘healthy housekeeping’. These sessions took place 1 month before the posttest and follow‐up test, respectively. The research team scheduled these sessions at specific times in selected health centres to minimise attrition rates. Furthermore, after the follow‐up test, participants were provided with a ‘Menopausal Self‐care’ booklet to enhance their knowledge about menopause and self‐care during this stage.

### 2.4 Statistical Analysis

The collected data were analysed using SPSS 27 software (Armonk, NY: IBM Corp). Data normality was assessed using the Kolmogorov‐Smirnov test. Descriptive statistics, including mean, standard deviation and frequency, were used. Inferential statistics, such as the chi‐square test, Cramer's V, and Phi coefficients, were applied. Moreover, independent *T*‐tests and Cohen's d were performed. Finally, Repeated Measures ANOVA and partial eta squared (η**²**) were used to analyse the data. To assess the relationship between constructs of the PRECEDE‐PROCEED model after intervention among the intervention group, PLS‐path modelling in SmartPLS version 3.2.8 was utilised as it is considered one of the top options for small sample sizes [16]. For all statistical tests, a p‐value below 0.05 was considered statistically significant.

## 3 Results

The descriptive findings of this study revealed statistically significant differences in the mean age (years) of the husbands between the control group (*N* = 62; *M* = 62.50, SD = 5.87) and the intervention group (*N* = 67; *M* = 59.86, SD = 5.78) [*t* (127) = –2.56, *p* = 0.01]. In addition, there were no statistically significant differences in the mean menopausal age (years) between the control group (*M* = 49.07, SD = 3.59) and the intervention group (*M* = 49.27, SD = 3.50) [*t* (138) = 0.33, *p* = 0.73].

Table 2 provides a summary of the frequency distribution of demographic and reproductive data in both the control and intervention groups.

**Table 2:** Frequency of demographic and reproductive characteristics of the participants in the study groups.

Characteristics	Control group		Intervention group		Chi‐square test		
	*n* 1	%^2^	*n* 1	%^2^	*X* 2 (df)	*p* value	Effect size
**Education**					2.59 (3)	0.45	0.13^a^
Elementary	8	11.4	9	12.9			
Middle–high school	17	24.2	25	35.7			
Diploma	32	45.7	26	37.1			
University degree	13	18.6	10	14.3			
**Marital status**					4.65 (2)	0.09	0.18^a^
Married	60	85.7	67	95.7			
Divorced	2	2.9	0	0			
Widowed	8	11.4	3	4.3			
**Occupation**					1.10 (2)	0.57	0.08^a^
Employed	5	7.1	3	4.3			
Homemaker	59	84.3	58	82.9			
Retirement	6	8.6	9	12.9			
**Husband's education**					4.87 (3)	0.18	0.18^a^
Elementary	6	8.5	8	11.4			
Middle–high school	13	18.6	14	20			
Diploma	38	54.3	26	37.1			
University degree	13	18.6	22	31.4			
**Husband's occupation**					0.41 (2)	0.81	0.05^a^
Government employee	8	11.4	6	8.6			
Self‐employed	13	18.6	12	17.1			
Jobless	3	4.3	4	5.7			
Retirement	46	65.7	48	68.6			
**Adequacy of monthly family income**					1.03 (2)	0.59	0.08^a^
Less than expenses	31	44.3	37	52.9			
Equal to expenses	31	44.3	26	37.1			
More than expenses	8	11.4	7	10			
**History of delivery methods**							
Vaginal delivery^c^	53	75.7	59	84.3	1.11 (1)	0.29	−0.10^b^
Elective caesarean section^c^	13	18.6	11	15.7	0.05 (1)	0.82	0.03 b
Emergency caesarean section^c^	13	18.6	10	14.3	0.20 (1)	0.64	0.05^b^
**History of breastfeeding** c	63	90	67	95.7	0.96 (1)	0.32	−0.11^b^
**History of hormonal contraceptive methods**							
Oral contraceptive pills^c^	36	51.4	35	50	< 0.001 (1)	1	0.01^b^
Ampulla^c^	5	7.1	4	5.7	< 0.001 (1)	1	0.02^b^

*Note. N* = 140 (*n* = 70 in each control and intervention groups).

^1^ Reflects number of participants.

^2^ Reflects percentages of participants.

^a^ Reflects results of ‘Cramer's V’ for ‘chi‐square test’

^b^ Reflects results of ‘Phi’ for ‘chi‐square test’ with a 2 × 2 contingency table.

^c^ Reflects the number or percentage of participants answering ‘yes’ to this question.

A significant difference was observed between the two groups 2 weeks after the intervention on quality of life [*t* (138) = 2.04, *p* = 0.04], and menopausal self‐care [*t* (138) = 5.67, *p* < 0.001]. These significant differences were maintained between the two groups 4 months after the intervention on quality of life [*t* (138) = 1.93, *p* = 0.04], and menopausal self‐care [*t* (138) = 5.20, *p* < 0.001]. The women's health scores were also significantly different between the two groups at this time [*t* (138) = 2.24, *p* = 0.02] (Table 3).

**Table 3:** Comparison of quality of life, women's health, menopausal self‐car, and MHLC‐C mean scores between control and intervention groups.

Groups	Before intervention			2 weeks after intervention		4 months after intervention		Mean differences (95% CI) [4 months after Intervention – before intervention]	*F* (df)		*p* value	η²^b^	
	Mean		SD^a^	Mean	SD^a^	Mean	SD^a^						
Quality of Life													
Control	63.73		12.49	64.28	12.76	65.52	12.08	1.78 (−0.31, 3.88)	2.40 (1.73)		0.10	0.03	
Intervention	62.68		13.10	68.82	13.50	69.67	12.19	6.99 (4.06, 9.92)	21.45 (2)		**< 0.001**	0.23***	
Mean differences (95% CI)	−1.05 (−5.33, 3.22)			4.53 (0.13, 8.92)		4.15 (0.09, 8.21)							
t (138)	−0.48			2.04		1.93							
p	0.62			**0.04**		**0.04**							
Cohen's d	−0.08			0.34		0.34							
Women's Health													
Control	4.68		1.40	5.29	4.88	4.74	1.30	0.05 (−0.25, 0.36)	1.12 (1.08)		0.29	0.01	
Intervention	4.64		1.50	4.98	1.74	5.27	1.50	0.63 (0.28, 0.98)	9.81 (2)		**< 0.001**	0.12**	
Mean differences (95% CI)	−0.04 (−0.53, 0.44)			−0.30 (−1.53, 0.91)		0.53 (0.06, 1)							
t (138)	−0.18			−0.49		2.24							
p	0.85			0.62		**0.02**							
Cohen's d	−0.03			−0.08		0.37							
Menopausal self−care													
Control	118.45		14.88	118.95	15.72	119.72	13.91	1.27 (−2.38, 4.93)	0.41 (2)		0.65	0.006	
Intervention	122.01		13.31	133.61	14.82	132.24	14.51	10.22 (7.43, 13.02)	50.84 (2)		**< 0.001**	0.42***	
Mean differences (95% CI)	3.55 (−1.16, 8.27)			14.65 (9.54, 19.76)		12.51 (7.76, 17.26)							
t (138)	1.49			5.67		5.20							
p	0.13			**< 0.001**		**< 0.001**							
Cohen's d	0.25			0.95		0.88							
Internal health locus of control													
Control	9.55	1. 90		8.44	2.42	8.78	2.22	−0.77 (−1.46, −0.08)	8.60 (2)	**< 0.001**			0.11**
Intervention	9.47	2.45		9.51	1.91	10.22	1.60	0.75 (0.06, 1.58)	3.97 (1.61)	**0.03**			0.05*
Mean differences (95% CI)	−0.08 (−0.82, 0.64)			1.07 (0.34, 1.80)		1.44 (0.79, 2.09)							
t (df)	−0.23 (138)			2.89 (130.90)		4.39 (125.59)							
p	0.81			0.004		< 0.001							
Cohen's d	−0.03			0.49		0.74							
Chance health locus of control													
Control	13.20	4.41		12.87	4.18	13.85	5.37	0.65 (−0.76, 2.08)	2.04 (1.75)	0.14			0.02
Intervention	13.97	5.88		12.75	5.28	12.77	5.18	−1.20 (−2.90, 0.50)	2.28 (1.53)	0.11			0.03
Mean differences (95% CI)	0.77 (−0.96, 2.51)			−0.11 (−1.70, 1.48)		−1.08 (−2.85, 0.68)							
t (df)	0.87 (128)			−0.14 (138)		−1.21 (138)							
p	0.38			0.88		0.22							
Cohen's d	0.14			−0.02		−0.20							
Doctor health locus of control													
Control	15.15	2.38		13.88	2.82	14.31	2.64	−0.84 (−1.54, −0.14)	8.09 (2)	< 0.001			0.10**
Intervention	14.94	2.72		14.78	2.06	15.44	2.11	0.50 (−0.31, 1.31)	2.62 (1.73)	0.08			0.03
Mean differences (95% CI)	−0.21 (−1.06, 0.64)			0.90 (0.07, 1.72)		1.12 (0.32, 1.92)							
t (138)	−0.49			2.15		2.79							
p	0.62			**0.03**		0.006							
Cohen's d	−0.08			0.36		0.47							
Others health locus of control													
Control	9.92	4.18		9.81	3.53	9.94	3.49	0.01 (−1.14, 1.17)	0.06 (1.73)	0.91			0.001
Intervention	10.75	4.18		10.70	4.03	10.88	4.02	0.12 (−1.08, 1.34)	0.06 (2)	0.93			0.001
Mean differences (95% CI)	0.82 (−0.57, 2.22)			0.88 (−0.38, 2.15)		0.94 (−0.31, 2.20)							
t (138)	1.17			1.38		1.48							
p	0.24			0.16		0.14							
Cohen's d	0.19			0.23		0.25							

*Note:* Mean parameter values for each of the analyses are shown for the control group (*n* = 70) and intervention group (*n* = 70), as well as the results of independent *t*‐test and Repeated Measures ANOVA comparing the parameter estimates between the two groups.

^a^ Reflects standard deviation.

^b^ Reflects partial eta squared.

** Reflects medium effect size.

*** Reflects large effect size.

In the intervention group, there were mean score differences in quality of life [*F* (2, 138) = 21.45, *p* < 0.001], women's health [*F* (2, 138) = 9.81, *p* < 0.001], and menopausal self‐care [*F* (2, 138) = 50.48, *p* < 0.001] at the pre‐test, posttest, and follow‐up stages. The effects of the intervention were maintained over time (Table 3).

A significant difference was found between the two groups 2 weeks after the intervention in relation to the Internal [*t* (130.90) = 2.89, *p* = 0.004] and Doctors [*t* (138) = 2.15, *p* = 0.03] subscales. Furthermore, these significant differences persisted between the two groups 4 months after the intervention on the Internal [*t* (125.59) = 4.39, *p* < 0.001] and Doctors [*t* (138) = 2.79, *p* = 0.006] (Table 3).

The findings showed that there was difference in the Internal subscale [*F* (1.61, 111.25) = 3.97, *p* = 0.03] across the three stages. In the control group, the mean scores for the Internal [*F* (2, 138) = 8.06, *p* < 0.001] and Doctors [*F* (2, 138) = 8.09, *p* < 0.001] subscales at the posttest and follow‐up stages were significantly lower than at the pre‐test stage (Table 3).

**Figure 3:**
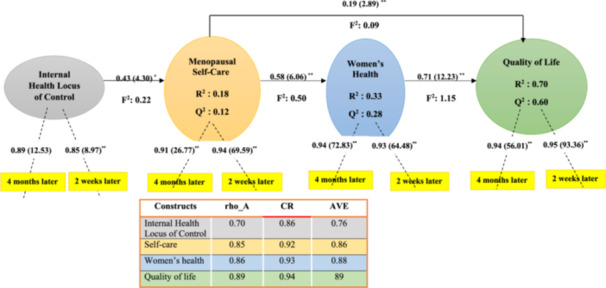
PLS (Partial Least Squares) estimates for the measurement model and structural model among intervention group. In each arrow, the first number indicates *b*‐value and the number in parentheses indicates *T*‐value. ∗*p* = 0.004, ∗∗*p* < 0.001.

As illustrated in Figure 3, the model fit indices – namely, the standardised root mean squared residual (SRMR) and the normed fit index (NFI) – were reported as 0.06 and 0.71, respectively. These values indicate a satisfactory model fit, as SRMR values below 0.10 or 0.08 and NFI values between 0 and 1 are generally considered acceptable [16]. To further validate the constructs of the PRECEDE‐PROCEED model after the intervention, a bootstrap approach was utilised. The model revealed that internal health control significantly impacts menopausal self‐care (*p* = 0.004), which in turn affects both women's health and their quality of life. This finding aligns with the direct effect of women's health on quality of life (*p* < 0.001).

To evaluate predictive validity, the blindfolding method was used, resulting in *Q*² values of 0.18 for menopausal self‐care, 0.33 for women's health, and 0.70 for quality of life. These results indicate the model possesses strong predictive relevance for the endogenous constructs. Predictive accuracy was also examined using R² values, which showed that internal health control accounted for 12% of the variation in menopausal self‐care. Moreover, menopausal self‐care explained 28% of the variation in women's health, and both menopausal self‐care and women's health together accounted for 60% of the variation in quality of life. These outcomes suggest that the model has substantial explanatory power.

Effect size (*F*²) was also calculated to determine the strength of relationships between variables. The *F*² value for the relationship between internal health control and menopausal self‐care was 0.22, indicating a moderate effect. The effect sizes for the links between menopausal self‐care and women's health, and between menopausal self‐care and quality of life, were 0.50 (large effect) and 0.09 (small effect), respectively. Notably, the relationship between women's health and quality of life had an *F*² of 1.15, signifying a very large effect, as detailed in Figure 3.

Finally, the study assessed the reliability and validity of the constructs using rho_A, composite reliability (CR), and average variance extracted (AVE). The rho_A and CR values exceeded 0.70 and 0.60, respectively, indicating that the measures are reliable. Likewise, the AVE values for all constructs were above the recommended threshold of 0.50, supporting the validity of the constructs. These findings are also presented in Figure 3.

## 4 Discussion

Training based on Longwe's empowerment framework and a community‐based participatory approach intervention significantly increased mean scores for quality of life, health, self‐care, and internal health locus of control. Similarly, Kafaei‐Atrian et al. reported a significant improvement in mean quality of life scores after implementing Longwe's empowerment framework for self‐care among menopausal women [17]. The similarity in the training topic, framework used for intervention, number and timing of tests after intervention may explain the similarity in the results obtained. Therefore, empowering women can improve their quality of life and help health professionals provide better education on self‐care for menopausal women [17]. Additionally, a study conducted by Hossein Mirzaee Beni et al. demonstrated a significant increase in quality of life and self‐care among postmenopausal women through self‐care training involving group discussion and interactive lectures. These findings suggest that active self‐care training is practical in significantly improving the quality of life and self‐care of postmenopausal women. It can be used to enhance outcomes related to the health of postmenopausal women [12]. Also, Seib et al. conducted a lifestyle programme for menopausal women with breast and gynaecological cancer. The findings showed improvements in general health and health‐related quality of life (HRQOL) scores. Therefore, considering how lifestyle factors such as physical activity, a healthy diet, stress control, and menopause management interact to influence health behaviour, interventions that address the interplay of multiple health behaviours have the potential for immediate and sustainable change [33]. Furthermore, the study on theory‐based educational programmes by Chair et al. demonstrated a significant increase in the mean scores of physical activities, dietary behaviour, body weight, body mass index, waist circumference, blood pressure, and Framingham risk among postmenopausal women. Therefore, a theory‐based educational programme may improve health behaviour scores and cardiovascular health outcomes [4].

In contrast, Monfaredi et al. reported no significant improvement in the quality of life and sleep of postmenopausal women after an Acceptance and Commitment Therapy (ACT) intervention in the form of counselling [26]. This difference can be attributed to the focus of their study on changing knowledge and attitudes, whereas the current study emphasises increasing empowerment. Empowerment interventions may prove more effective than simply altering knowledge and attitudes [27]. Additionally, the study by Rindner et al. on the impact of group education and person‐centred support on mental health and quality of life in menopausal women revealed that while long‐term improvements in health‐related quality of life and reductions in mental, urogenital, and stress‐related symptoms were not sustained in the group education setting, they were maintained in the person‐centred support group. Therefore, tailored education can effectively enhance women's health during menopause and potentially prevent the development of menopausal symptoms and problems [26].

This study introduces a transparent, theory‐based, equity‐focused, and community‐based participatory approach. Its practical findings can be utilised as training programmes for various health centres with similar characteristics. Another strength of this study is that its findings are applicable to all women, with a focus on empowering them.

Although the current research has several strengths, this study also had several limitations that need to be addressed. First, the reliance on self‐reported data collection and lack of blinding, which are common limitations in many studies, raises questions about the accuracy and reliability of the findings. Additionally, we only considered urban health centres and literate women of a specific age. Therefore, it is recommended that further research be conducted to address these limitations, particularly in populations with diverse social, economic, and cultural backgrounds. This is important because various factors, such as environmental, social, biological, cultural and psychological factors, can impact the understanding of menopause [6, 9, 31]. Furthermore, the health centres lacked educational technology for PowerPoint presentations, movies, video clips, audio podcasts, or hearing aids. Also, we considered a 4‐month follow‐up period. Further research is recommended using technology in education and extending the follow‐up period. These methods have the potential to improve people's learning and understanding of the persistence of outcomes and results [22]. Ultimately, while intention to treat (ITT) is ideal for RCTs, in this educational behavioural context with missing outcome data, a per‐protocol analysis was applied to evaluate the actual effectiveness of the intervention [1].

## 5 Conclusion

Training programmes based on Longwe's empowerment framework and a community‐based participatory approach have the potential to enhance individuals' quality of life, health, self‐care, and perceived internal health control. The outcomes of this intervention can provide valuable insights for health policymakers considering the implementation of community‐based participatory approaches to education, public awareness, and accessibility, ultimately empowering individuals to adopt healthier behaviours. Furthermore, healthcare professionals can use this programme as an educational tool to motivate behaviour and lifestyle changes in clients seeking to improve their health habits. It is also essential for future research to explore the potential impact of this training programme on the empowerment of women of different age groups, socioeconomic backgrounds and cultural contexts.

## Author Contributions

**Khadijeh Khademi:** conceptualization, investigation, methodology, software, data curation, formal analysis, writing – orginal draft, and writing – review and editing. **Mohammad Hossein Kaveh:** supervision, conceptualization, methodology, validiation, formal analysis, and writing – review and editing. **Mahin Nazari:** conceptualization, methodology, resources, visualization, and project administration. **Abdolrahim Asadollahi:** methodology, formal analysis, software, data curation, and writing – review and editing. All authors read and approved the final manuscript.

## Ethics Statement

This study was conducted under the clinical trials registration number IRCT20230716058792N1 which was registered on 14/08/2023 on the Iranian Registry of Clinical Trials (IRCT) and approved by the Ethics Committee of Shiraz University of Medical Sciences (IR. SUMS. SCHEANUT. REC.1402.049). According to the Declaration of Helsinki, all the participants were required to complete a written informed consent form.

## Conflicts of Interest

The authors declare no conflicts of interest.

## Data Availability

The datasets used and/or analysed during the current study are available from the corresponding author on reasonable request.
